# Defining Risk-Based Monitoring Frequencies to Verify the Performance of Water Treatment Barriers

**DOI:** 10.1021/acs.estlett.3c00154

**Published:** 2023-03-13

**Authors:** Émile Sylvestre, Eva Reynaert, Timothy R. Julian

**Affiliations:** † Eawag, Swiss Federal Institute of Aquatic Science and Technology, Dübendorf CH-8600, Switzerland; ‡ ETH Zürich, Institute of Environmental Engineering, 8093 Zürich, Switzerland; § Swiss Tropical and Public Health Institute, 4051 Basel, Switzerland; ∥ University of Basel, 4055 Basel, Switzerland

**Keywords:** Quantitative microbial risk assessment (QMRA), failure analysis, drinking water, water reuse, online monitoring, log reduction value (LRV) verification

## Abstract

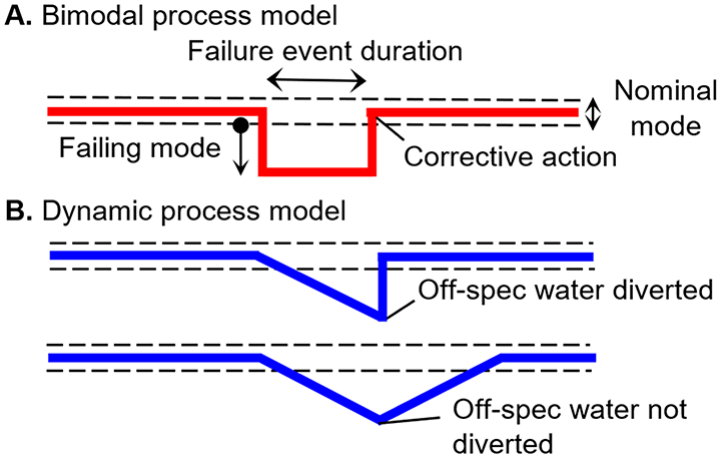

Preventing failures of water treatment barriers can play an important role in meeting the increasing demand for microbiologically safe water. The development and integration of failure prevention strategies into quantitative microbial risk assessment (QMRA) offer opportunities to support the design and operation of treatment trains. This study presents existing failure models and extends them to guide the development of risk-based operational monitoring strategies. For barriers with rapid performance loss, results show that a failure of 15 s should be reliably detected to verify a log reduction value (LRV) of 6.0; thus, detecting and remediating these failures may be beyond current technology. For chemical disinfection with a residual, failure durations in order of minutes should be reliably detected to verify a LRV of 6.0. Short-term failures are buffered because the disinfectant residual concentration sustains a partial reduction performance. Therefore, increasing the contact time and hydraulic mixing reduces the impact of failures. These findings demonstrate the importance of defining precise frequencies to monitor barrier performances during operation. Overall, this study highlights the utility of process-specific models for developing failure prevention strategies for water safety management.

## 1 Introduction

Reduction of enteric pathogen concentrations in environmental and recycled waters is required to control human health risks associated with acute potable and nonpotable water exposures. Effective water treatment barriers must be designed and operated to ensure adequate pathogen reduction. The performance of these barriers, usually expressed in log_10_ reduction values (LRVs), can be attributed based on validation testing and verification during operation.

For LRV *validation*, performance data are obtained by measuring the count of spiked or naturally occurring microorganisms in water samples collected at the inflow and outflow of the water treatment process.^1^ These demonstrations are usually undertaken at bench-, pilot-, or full-scale under nominal operating modes. However, water treatment barriers can fail during operation, and failure events can substantially reduce the average treatment performance over the long term if no corrective actions are taken.^2,3^ Therefore, LRV *verification* is usually performed during operation by checking operational limits of process parameters (e.g., turbidity, disinfectant concentration).^4^ The probability of detecting a failure event depends on the sensitivity of the process parameters and their monitoring frequencies.

The duration, frequency, and magnitude of failure events are typically unknown, but their impacts on treatment performance can be evaluated theoretically. A bimodal process model was formulated by Teunis and Havelaar (1999)^3^ to predict the average probability of passage of a microorganism through a treatment barrier operating in either nominal or failure modes. Teunis et al. (2004)^5^ generalized the model for multiple barriers operating independently in series. In a subsequent study by Smeets et al. (2010),^6^ the bimodal process model was used to demonstrate that the operational monitoring frequency influences a system’s guaranteed LRV compliance. Therefore, monitoring should be adapted to align with the magnitude of the system’s desired LRV.^6^

More recently, Pecson et al. (2017)^7^ developed a Monte Carlo method to evaluate the treatment redundancy (i.e., performance beyond the minimum needed) required in a direct potable reuse treatment train to buffer out the impact of selected failure durations per barrier. They chose 15 min as a reasonable minimum interval for operational monitoring. For a 15 min 6.0-log reduction failure occurring once per year, excess log capacities to comply with annual and daily risk targets are about 2.0 and 4.0, respectively. Including such excess log capacities in reduction targets set for some water treatment trains, such as those currently in development for onsite nonpotable water reuse, could pose challenges in terms of implementation.

Further development and integration of failure prevention strategies into a broader quantitative microbial risk assessment (QMRA) context offer opportunities for the design of treatment trains and operational monitoring strategies. The objectives of this study are (i) to present failure models for a single barrier and multiple barriers in series, (ii) to expand the single barrier model to a use case of the failure of a chemical disinfectant dosing pump, and (iii) to use these models to establish relationships between failure durations, the validated and the verified LRVs of barriers.

## 2 Materials and Methods

### 2.1 Bimodal Process Model for a Single Barrier

The simplest model that can be formulated to describe the performance of a treatment process working in two modes is the bimodal distribution.^5^ For a bimodal performance characterized by a probability of failure (*p*_*f*_), the distribution of the probability of passage of a microorganism (π) is given by
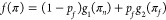
1where, *g*_1_ (*π*_*n*_) and *g*_2_ (*π*_*f*_) are, respectively, the probability distributions of the passage of the organism in nominal and failure modes. For QMRA, the performance of a treatment process can be summarized by its expected value, i.e., arithmetic mean probability of passage.^8^ The expected value of *f* (π) can be calculated as follows:
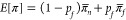
2where *π*_*n*_ and *π*_*f*_ are the arithmetic means of *g*_1_ (*π*_*n*_) and *g*_2_ (*π*_*f*_), respectively.

### 2.2 Dynamic Process Model for Chemical Disinfection

The bimodal process model assumes that failures develop abruptly, but for some treatment processes, the performance in failure mode may degrade over time. One example is the failure of a chemical dosing pump. Following the pump failure, the disinfectant concentration in the contact zone can be reduced below the operational limit due to dilution. However, depending on the hydraulic mixing conditions, the disinfectant residual concentration can sustain a partial reduction performance for a certain period.

As a starting point to formulate this model, the two extremes of hydraulic mixing conditions**—** the plug flow reactor (PFR) and the continuous flow stirred tank reactor (CSTR)**—**can be examined. In a perfect PFR (e.g., serpentine tubing), the water is not mixed or dispersed in the contact zone. Therefore, the contact between the disinfectant and organisms immediately stops when the failure occurs, and the process model is bimodal. For an ideal CSTR (e.g., unbaffled tank), complete mixing is assumed in the contact zone; thus, degradation of the inactivation performance is expected during the failure.

In reality, hydraulic mixing during chemical disinfection is neither an ideal PFR nor an ideal CSTR. A common approach to predict disinfection in a nonideal reactor (e.g., a baffled tank) is to use a CSTR-in-series model.^9^ This model predicts the arithmetic mean probability of passage of viable organisms as follows:
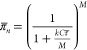
3where *k* is a first-order kinetic rate constant, *C* the disinfectant concentration in the contact zone, τ the *total* mean hydraulic residence time (HRT) in the series of CSTRs, and *M* the number of CSTRs in series. The parameter *M* can be selected to predict poor hydraulics (two CSTRs), medium-good hydraulics (six CSTRs), and near-perfect plug flow (20 CSTRs).^10^

#### t

In failure mode, the mean disinfectant concentration in the contact zone at a time has the simple exponential form:^11^
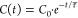
4where *C*_0_ is the initial disinfectant concentration. A dynamic CSTR-in-series model can be formulated by substituting *C* in eq 3 with eq 4. The momentary probability of passage is then
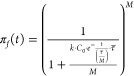
5

This equation simplifies the underlying dynamic process as it assumes that, at a time *t* after the pump failure, the disinfectant concentration in each CSTR is equal to the one in the first CSTR. This model provides a conservative estimate of the performance because it models disinfectant concentrations below expected concentrations. The arithmetic mean probability of passage during this failure mode (*π*_*f*_) was numerically approximated by averaging a series of *π*_*f*_ computed at time steps of 1 s for the duration of the failure event.

If it is assumed that the treatment performance instantaneously returns to *π*_*n*_ when the failure is detected, then *E*(π) can be calculated using eq 2. For practice, this means that the off-spec water (i.e., treated water that does not meet specifications) is diverted until the disinfectant concentration in the system returns to *C*_0_.

If off-spec water is not diverted, the failure event will last until the disinfectant concentration returns to *C*_0_. The impact of the treatment recovery on the average performance can be included in the model as follows:^6^
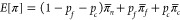
6where *p*_*c*_ is the proportion of time required for correction, and *π̅*_*c*_ is the average probability of passage during the corrective action. If the dosing rate of the pump is the same in nominal and recovery modes, *E*[π] can be calculated as follows:
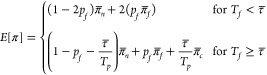
7where *T*_*f*_ is the duration of the failure event, *T*_*p*_ the total duration of the reference time period, and *π̅*_*c*_ is given by eq 5 for *t* = τ.

### 2.3 Multiple Barriers in Series Operating Independently

The bimodal process model can be generalized for a combination of treatment barriers operating independently in series.^5^ For a given *p*_*f*_, the probability of getting *K* failing barriers out of *N* barriers is given by the binomial distribution, and the expected value of *f*(π) for *N* combined barriers becomes
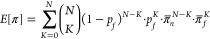
8

This generalized bimodal process model can be understood as follows: *K* barrier failures occur with probability *p*_*f*_^*K*^, *N* – *K* barriers operating nominally with probability (1 – *p*_*f*_)^*N*−*K*^, and the binomial coefficient  accounts for the different combinations that *K* barriers can fail. This model can also be generalized to treatment trains having barriers with different probabilities of failure and performances in nominal and failure modes. For instance, the expected value of *f*(π) for a barrier *i* and a barrier *j* in series becomes
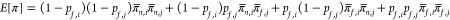
9

Equation 9 can also be used to evaluate the performance of a single barrier having two failure modes, such as a UV/H_2_O_2_ advanced oxidation process, in which both the H_2_O_2_ dosing pump and the UV process can have distinct failure rates.

For multiple barriers in series, if the failure duration of each barrier is short, the probabilities of simultaneous barrier failures have negligible influence on the performance of combined barriers.^7,12^ Therefore, the expected value of *f*(π) for a total of *N* barrier *k* in series, *E*[π]_*T*_, can be estimated by
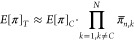
10where *E*[π]_*C*_ is the expected probability of passage (verified LRV) of the critical barrier in the treatment train, which can be determined as follows:
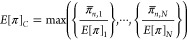
11

### 2.4 Simulations

#### 2.4.1 Momentary Reduction Performances

Momentary reduction performances of water treatment barriers were illustrated by two examples. First, momentary reduction performances predicted by the bimodal process model were represented using cumulative distribution functions (CDFs) for treatment trains with 1–3 barriers operating independently in series. For each barrier, *π*_*n*_ and *π*_*f*_ were set to 2.0-log and 0.5-log, respectively, and *p*_*f*_ was set to 10%. Second, momentary inactivation performances following a disinfectant dosing pump failure were illustrated for ratios of *T*_*f*_ to τ. Hydraulic conditions for series of two, six, and 20 CSTRs in series were selected, as defined by Petterson and Stenström (2015),^10^ and *π*_*n*_ was set to 4.0-log.

#### 2.4.2 Performance Verification Curves

Performance verification curves were simulated with the bimodal and dynamic process models. These curves predict the verified LRV from a validated LRV and a *p*_*f*_. The following assumptions were made:

1. *T*_*p*_ is one year or one day.
2. *T*_*f*_ includes the time to detect, validate, and respond to the failure event.
3. For the bimodal process model, *π*_*f*_ is 0.0-log, and the system instantaneously recovers to *π*_*n*_ at *T*_*f*_.
4. For the dynamic process model, the disinfectant concentration is measured at the point at which the contact time is achieved. The system instantaneously or gradually recovers to *π*_*n*_ at *T*_*f*_.

R codes used to generate figures are provided in the Supporting Information.

## 3 Results and Discussion

**Figure 1:**
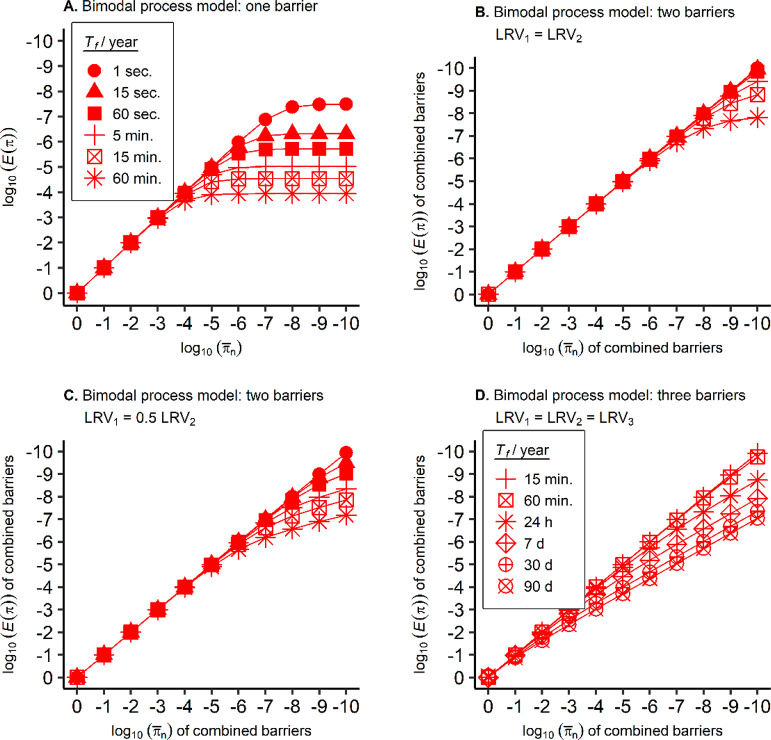
Performance verification curves simulated with the bimodal process model. Verified performances are shown for different nominal performances and *T*_*f*_ per year. Simulations are for (A) one barrier, (B) two independent barriers in series with the same validated LRV and *p*_*f*_, (C) two independent barriers in series having the same *p*_*f*_ but different validated LRVs, (D) three independent barriers in series with the same validated LRV and *p*_*f*_. Note that axes show validated and verified LRVs for combined barriers in B, C, and D.

Failure prevention strategies can be developed to control the impact of treatment failures on microbial water quality. A general finding, illustrated in this work and by others,^7^ is that process independence can be assumed when treatment trains are composed of multiple independent barriers in series and failure probabilities are low (Figure 1). As a result, the microbial risk associated with failure events is driven by a single critical barrier in the treatment train. The failure duration, frequency, and magnitude of this barrier will impact the risk.

The failure duration of a barrier can be minimized by rapidly detecting failures to remediate them. Operational parameters of water treatment barriers can often be monitored at high frequencies; however, to verify a LRV credit of 6.0, as granted by many regulatory agencies,^13−15^ and comply with an annual risk target, failure durations of 10 s per year need to be controlled for a barrier with rapid loss in reduction performances (Figure 1A). The control of such short failure durations may be beyond current monitoring technology. T90 response times of water quality sensors (i.e., the time required to reach 90% of the final value following an abrupt change) can vary from a few seconds to tens of seconds.^16^ Other jurisdictions are granting a maximum LRV credit of 4.0,^4^ which can be verified by controlling a failure of 15 min per year. More precise definitions of minimum operational monitoring frequencies could help develop risk-based standards. The terms “online” or “continuous” monitoring are often stated in guidelines and regulations, but it is unclear what they mean with regard to monitoring frequency. These terms involve subjectivity and can introduce biases in the assessment of treatment performances.

**Figure 2:**
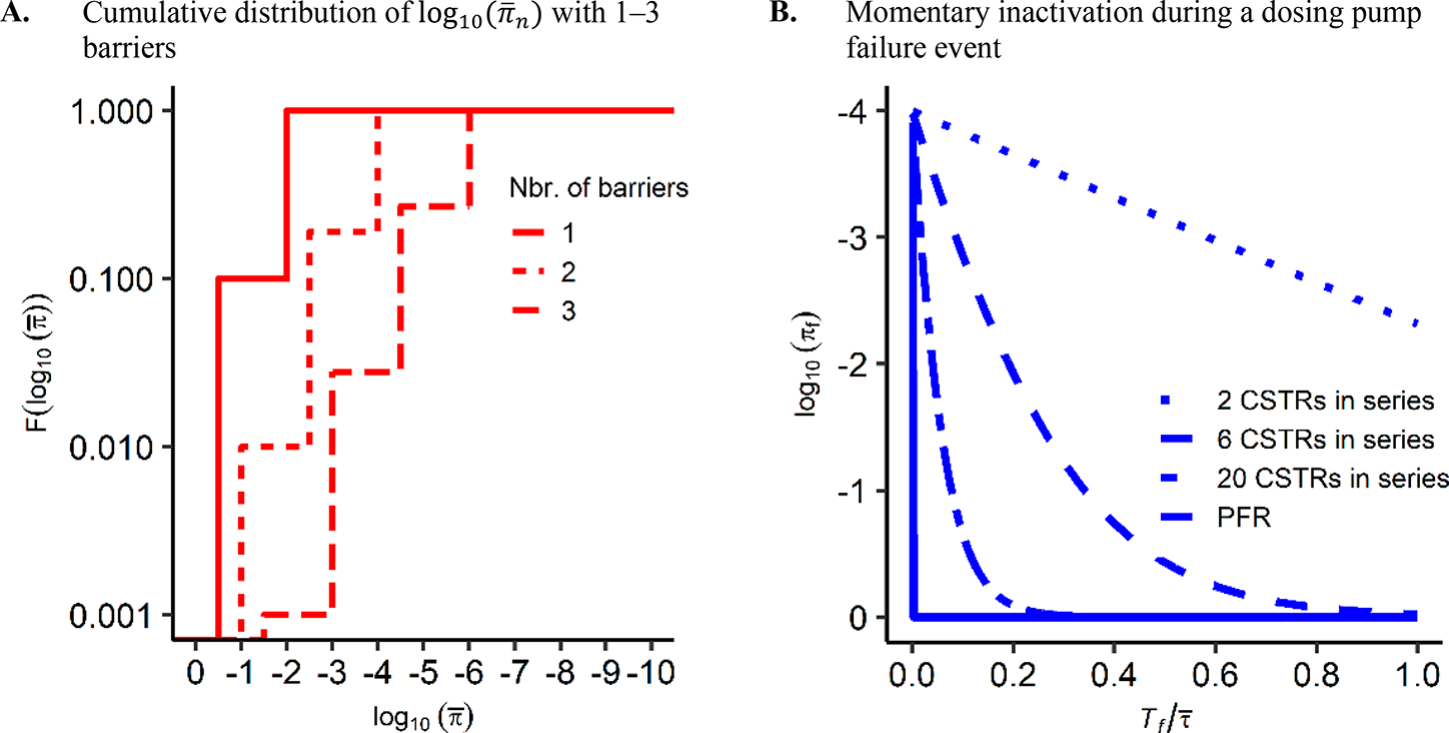
(A) Simulated cumulative distributions of the performance for treatment trains with 1–3 barriers operating independently in series. For each barrier, *π*_*n*_ = 0.01 (LRV of 2.0), *π*_*f*_ = 0.3 (LRV of 0.5), and *p*_*f*_ is 10% of its operational time (adapted from with permission from ref (5)). (B) Simulated momentary inactivation performances of a disinfection process following the failure of a disinfectant dosing pump for a PFR, two CSTRs, six CSTRs, and 20 CSTRs in series. Inactivation performances are shown for ratios of *T*_*f*_ to the mean τ, and *π*_*n*_ = 0.0001 (LRV of 4.0). Note that the *p*_*f*_ was not set to 10% to represent a realistic scenario but to improve visualization. Figure S3 illustrates cumulative distributions for different *p*_*f*_ and *π*_*f*_.

**Figure 3:**
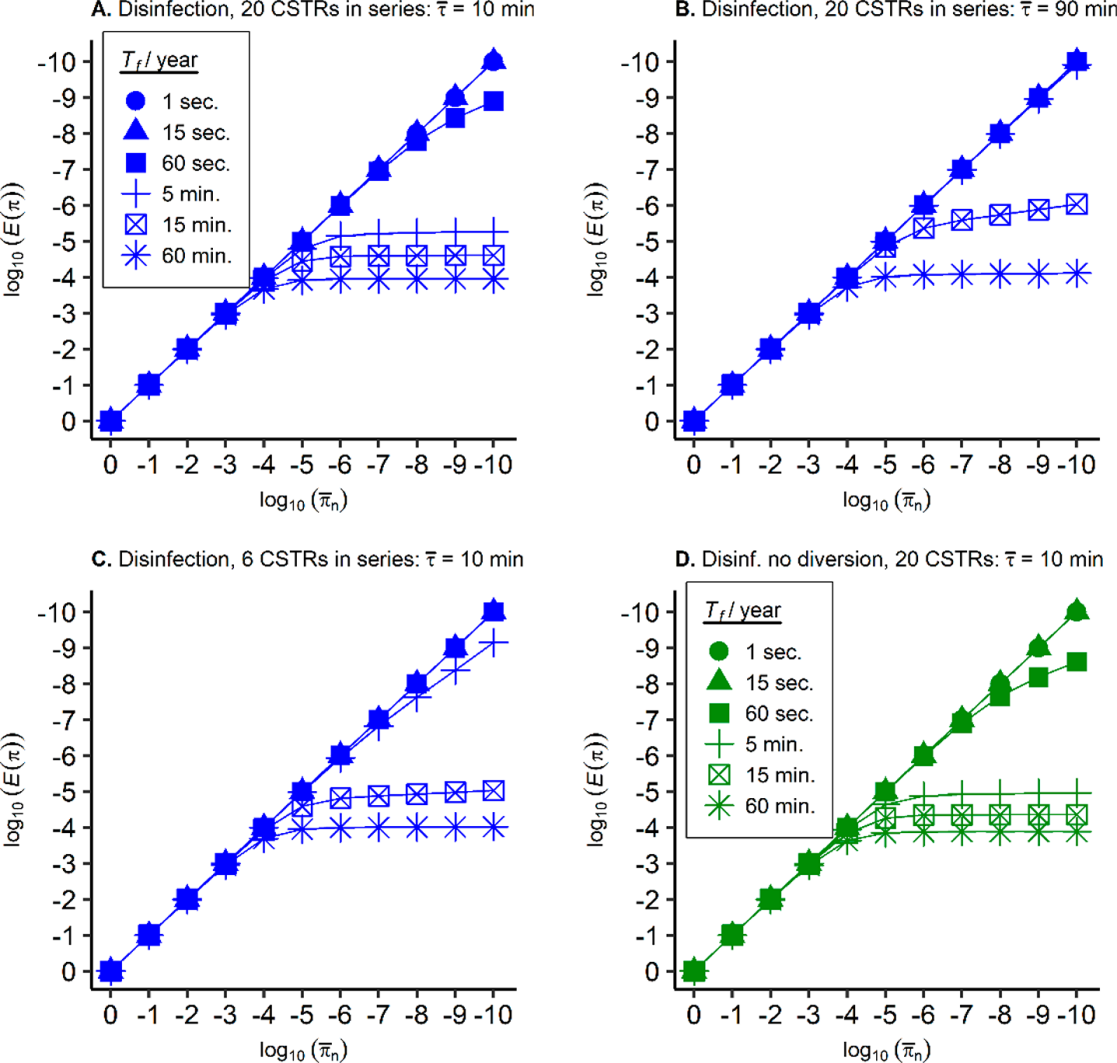
Performance verification curves simulated with the dynamical process model for the failure of a chemical disinfectant dosing pump. Verified performances are shown for different nominal performances and *T*_*f*_ per year. Simulations are for (A) 20 CSTRs in series with τ of 10 min with diversion of off-spec water, (B) 20 CSTRs in series with a τ of 90 min with diversion of off-spec water, (C) six CSTRs in series with a τ of 10 min with diversion of off-spec water, and (D) 20 CSTRs in series with a τ of 10 min without diversion of off-spec water.

Failure prevention strategies can also be developed by minimizing failure magnitudes (i.e., loss in treatment), for example, by setting operation limits to allow corrective actions to be taken during a partial treatment loss.^6^ Simulation results for chemical disinfection with a residual reveal that LRV credit of 6.0 can be verified to comply with annual and daily risk targets when failure durations in order of minutes can be detected (Figure 3, Figure S2). Failure durations of 60 s or less have a negligible influence on the verified LRV in near-perfect plug flow conditions (20 CSTRs) for an HRT of 10 min (Figure 2B, Figure 3A). Increasing the HRT and hydraulic mixing increases the buffering effect (Figure 3B, C), and diverting off-spec water during the restart of a dosing pump has a negligible impact on the verified LRV (Figure 3A, D).

Therefore, a chemical disinfection system with a disinfectant residual can be designed and monitored to verify high LRVs without diverting off-spec water. It is noteworthy that model-based and signal-based methods exist for fault detection within the hydraulic and mechanical parts of pumps.^17^ These methods could detect failures faster than checking disinfectant concentration limits. Extending this model to account for the decline of the disinfectant concentration in each CSTR over time could help investigate the degree to which hydraulics impact LRV verification.

This study emphasizes that the performance of a water treatment barrier is defined not only by its nominal performance but also by undetected failure events. It is insufficient to account for validated LRVs; LRVs also need to be verified to give a complete picture of expected microbial risks. This problem requires prompt attention as several risk-based standards are currently in development for water reuse. This work demonstrates that failure prevention strategies could aid in optimizing treatment train design and operational monitoring using QMRA. Providing redundancy by adding one or multiple barriers, as proposed for direct potable reuse,^7,12,18^ may be costly but can compensate for infrequent operational monitoring. Alternatively, more frequent operational monitoring and effective mitigation of failure events can reduce the excess log capacity needed for redundancy. Further research comparing the feasibility of these options could benefit water reuse implementation. The models presented in this study can be used and expanded to inform the development of treatment trains and operational monitoring strategies within a risk-based framework.

## Supporting Information Available

Figures S1–S3: Performance verification curves simulated for a daily risk target; additional simulated cumulative distributions of the performance for alternative combination of alternative visualizations of the impact of failure conditions on cumulative distributions of the performance of a treatment train (PDF)
R codes to replicate figures from the manuscript (ZIP)

## List of Symbols

*C*: Chemical disinfectant concentration
*E*[π]: Expected value of the probability of passage for a barrier
*E*[π]_*C*_: Expected value of the probability of passage for a critical barrier
*E*[π]_*T*_: Expected value of the probability of passage for a treatment train
*f*(π): Probability distribution of passage
*g*_1_ (*π*_*n*_): Probability distribution of passage in nominal mode
*g*_2_ (*π*_*f*_): Probability distribution of passage in failure mode
*k*: First-order kinetic rate constant
*K*: Number of failing barriers in treatment train
*M*: Number of continuous stirred tank reactors in series
*N*: Total number of barriers in treatment train
*p*_*c*_: Proportion of time required for correction
*P*_*f*_: Probability of failure
*π*_*c*_: Average probability of passage during corrective action
*π**®*_*n*_: Average probability of passage in nominal mode
*π*_*f*_: Average probability of passage in failure mode
*t*: Time after the failure of a disinfectant dosing pump
*T*_*f*_: Duration of the failure event
*T*_*p*_: Total duration of the reference time period
τ: Average hydraulic residence time
