# Supplementary material for: Defining Risk-Based Monitoring Frequencies to Verify the Performance of Water Treatment Barriers
Source: Environ Sci Technol Lett. 2023 Mar 13;10(4):379–84. doi: 10.1021/acs.estlett.3c00154 (PMC10100555; doi:10.1021/acs.estlett.3c00154)
Supplement: Supplementary file 1 — ez3c00154_si_001.pdf [file ez3c00154_si_001.pdf]

Supplementary information

Journal: Environmental Science & Technology Letters

Title: Defining risk-based monitoring frequencies to verify the performance of water treatment barriers

Authors: Émile Sylvestre, Eva Reynaert, Timothy R. Julian

Number of pages: 4

Number of figures: 3

Supplementary Figure 1: Additional performance verification curves simulated with the bimodal process model to comply with a daily health risk target rather than an annual health risk target.

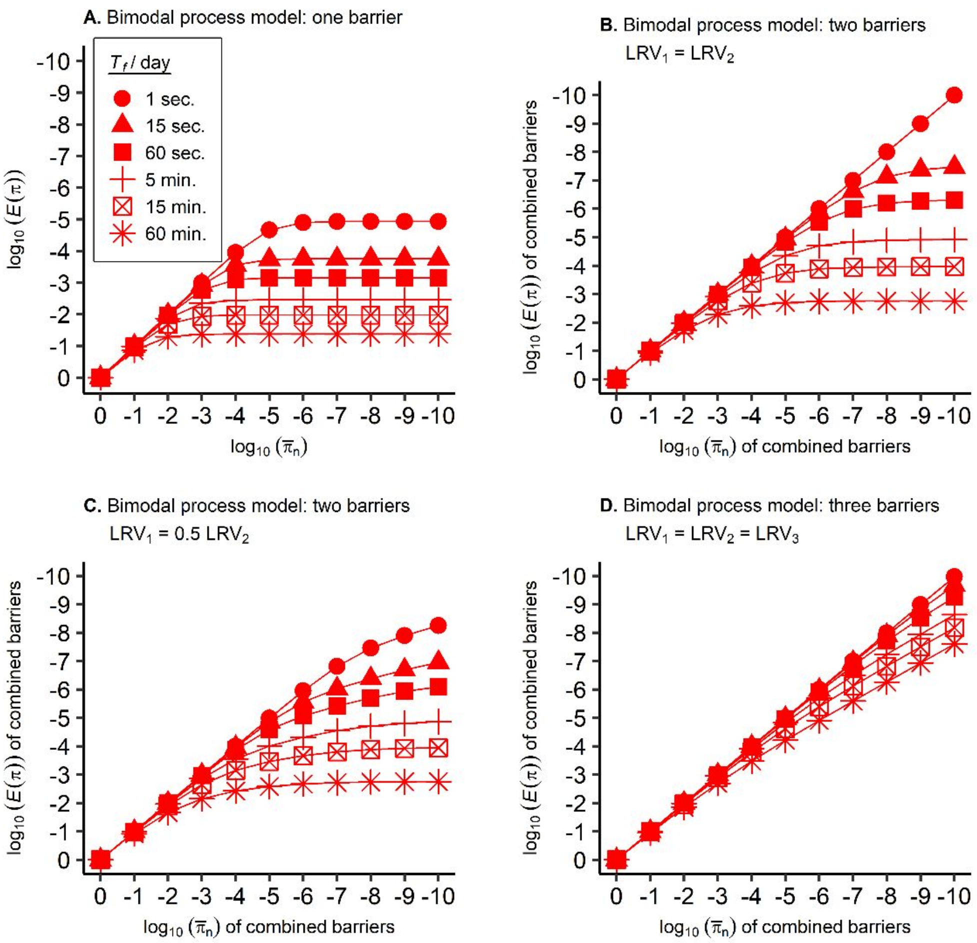

Fig. S1. Performance verification curves simulated with the bimodal process model for different nominal performances and T_f per day. Simulations are for: **A.** one barrier, **B.** two independent barriers in series with the same validated LRV and p_f, **C.** two independent barriers in series having the same p_f but different validated LRVs **D.** three independent barriers in series with the same validated LRV and p_f, **D.** Note that axes show validated and verified LRVs for combined barriers in B., C., D.

Supplementary Figure 2: Additional performance verification curves simulated with the dynamic process model to comply with a daily health risk target rather than an annual health risk target.

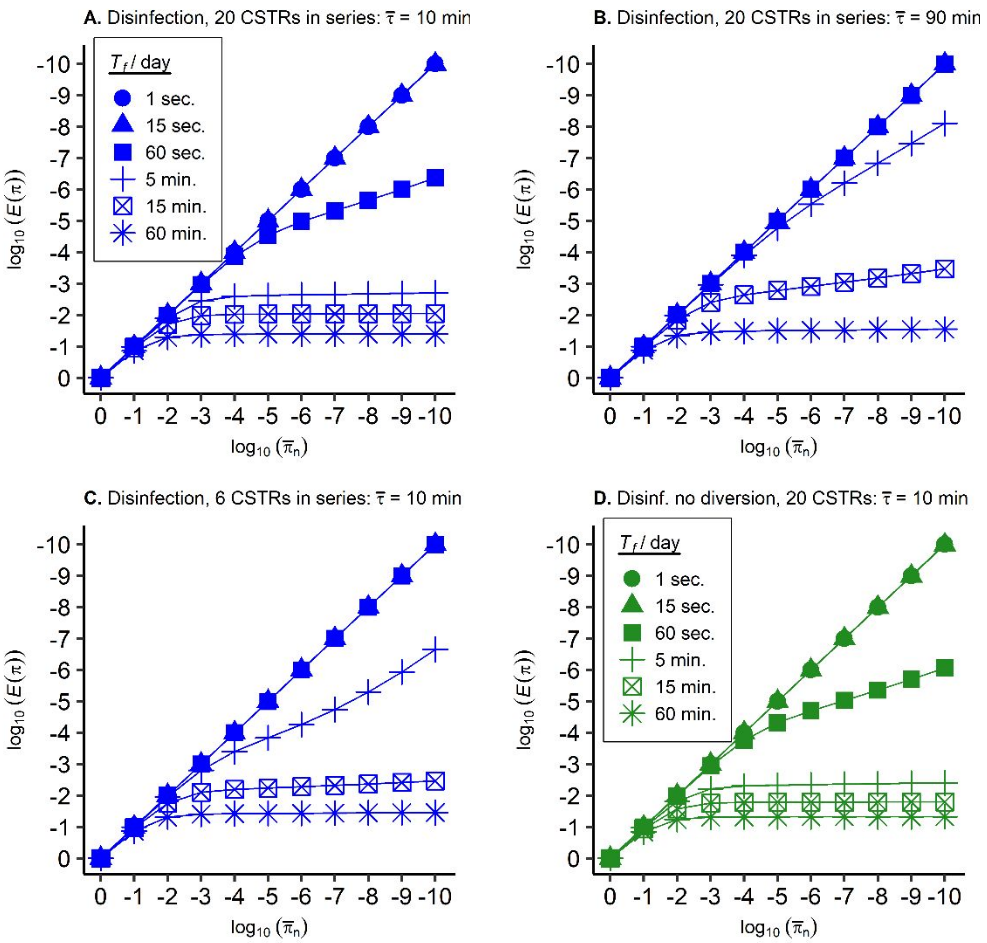

Fig. S2. Performance verification curves simulated with the dynamical process model for the failure of a chemical disinfectant dosing pump. Verified performances are shown for different nominal performances and T_f per year. Simulations are for: **A.** 20 CSTRs in series with $\bar{\tau}$ of 10 min with diversion of off-spec water. **B.** 20 CSTRs in series with a $\bar{\tau}$ of 90 min with diversion of off-spec water. **C.** 6 CSTRs in series with a $\bar{\tau}$ of 10 min with diversion of off-spec water. **D.** 20 CSTRs in series with a $\bar{\tau}$ of 10 min without diversion of off-spec water.

Supplementary Figure 3: Additional visualizations of the impact of the variation of failure conditions on cumulative distributions of the performance for treatment trains with 1–3 barriers operating independently in series.

A. **B.**

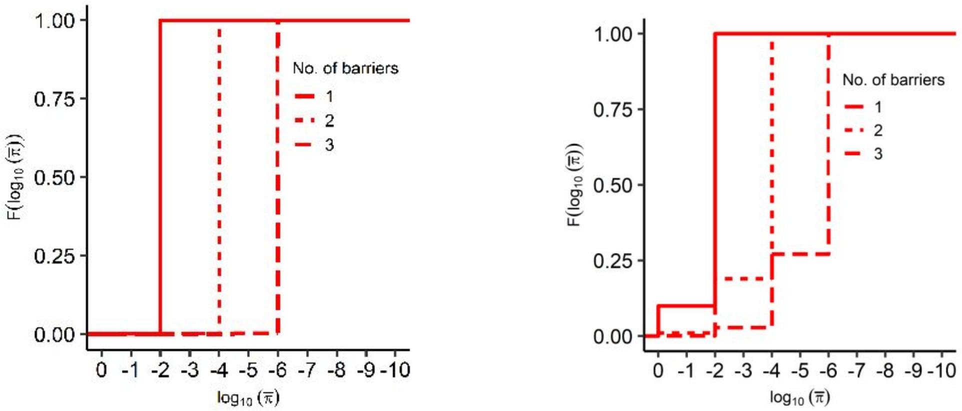

Fig. S3. Simulated cumulative distribution of the performance for treatment trains with 1–3 barriers operating independently in series. **A.** Parameters for each barrier are: $\bar{\pi}_n$= 0.01 (LRV of 2.0), $\bar{\pi}_f$= 0.3 (LRV of 0.5), and p_f is 0.1% of its operational time. **B.** Parameters for each barrier are: $\bar{\pi}_n$= 0.01 (LRV of 2.0), $\bar{\pi}_f$= 1.0 (LRV of 0.0), and p_f is 10% of its operational time.
