# Supplementary material for: Defining Risk-Based Monitoring Frequencies to Verify the Performance of Water Treatment Barriers
Source: Environ Sci Technol Lett. 2023 Mar 13;10(4):379–84. doi: 10.1021/acs.estlett.3c00154 (PMC10100555; doi:10.1021/acs.estlett.3c00154)
Supplement: Supplementary file 2 — ez3c00154_si_002.zip [file ez3c00154_si_002.zip › R_codes_Mar10/Description_supporting_information_file_R_codes.docx]

**Supplementary information: R codes**

**Journal:** Environmental Science & Technology Letters

**Title:** Defining risk-based monitoring frequencies to verify the performance of water treatment barriers

**Authors:** Émile Sylvestre, Eva Reynaert, Timothy R. Julian

**Number of R codes:** 11

**Description**: R codes can be used to replicate all figures from the manuscript.
